# Combination of Interleukin-15 With a STING Agonist, ADU-S100 Analog: A Potential Immunotherapy for Prostate Cancer

**DOI:** 10.3389/fonc.2021.621550

**Published:** 2021-03-10

**Authors:** Ana M. Esteves, Efthymia Papaevangelou, Prokar Dasgupta, Christine Galustian

**Affiliations:** ^1^Peter Gorer Department of Immunobiology, School of Immunology and Microbial Sciences, King’s College London, Guy’s Hospital, London, United Kingdom; ^2^Urology Centre, Guy’s Hospital, London, United Kingdom

**Keywords:** Interleukin-15, STING agonist, prostate cancer, NK cells, cytotoxicity

## Abstract

Prostate cancer is the second most commonly diagnosed cancer in men with mortality rates, overtaking those for breast cancer in the last 2 years in the UK. Despite advances in prostate cancer treatments, over 25% of men do not survive over 5 years with advanced disease. Due to the success of immunotherapies in treating other cancers, this treatment modality has been investigated for Prostate cancer, however, the sole FDA approved immunotherapy so far (Provenge™) only extends life by a few months. Therefore, finding immunotherapeutic agents to treat prostate cancer is of major interest. Our group has previously shown that Interleukin-15 (IL-15), unlike other therapeutic cytokines such as IL-2 and IL-12, can stimulate expansion and activity of CD8 T cells and NK cells *in vitro* when they are exposed to prostate cancer cells, while studies in mice have shown a 50% reduction in tumor size with no apparent toxicity. In this study, we aim to examine potencies of IL-15 in combination with a cyclic dinucleotide (CDN) that activates the Stimulator of Interferon-Gene (STING) receptor. Selected CDNs (also known as STING agonists) have previously been shown to activate both T cells and dendritic cells through STING. We hypothesize that the combination of STING agonists and IL-15 can additively increase NK and T cell activity as they act to increase type I interferons (IFNs) through STING activation and IFN-γ through IL-15. In prostate cancer-lymphocyte co-cultures we now show that combination of IL-15 and the STING agonist ADU-S100 analog induces a marked killing of cancer cells above that seen with IL-15 or ADU-S100 alone. We show that this is related to a potent activation of NK cells resulting in increased perforin and CD69 expression, and up to a 13-fold increase in IFNγ secretion in the co-cultures. NK cells are responsible for killing of the cancer cells, as shown by a lack of cytotoxicity in NK depleted lymphocyte-tumor cell co-cultures, or in co-cultures of B and T cells with tumor cells. In summary, we propose that the combination of IL-15 and the sting agonist ADU-S100 analog may be potently effective in treatment of prostate cancer.

## Introduction

The last decade has seen an increase in the number of immunotherapeutic agents tested for the treatment of solid tumors. However, progress made in the field of prostate cancer is less evident (1). The sole FDA-approved vaccine tested so far for the disease (Sipuleucel-T, also known as Provenge™) only extended patient’s life by 4 months: Provenge is an autologous dendritic cell preparation from cells taken from the patient and treated with a fusion protein consisting of Granulocyte-macrophage colony-stimulating factor (GMCSF) and the antigen prostate acid phosphatase (PAP) associated with prostate tumors. These cells, once injected back into the patients can then present antigen to prime T cells into becoming PAP antigen specific cytotoxic T cells directed against the patients tumors (2–4). A recent publication revealed that prostate cancer is still the second leading cause of cancer mortality among males in the USA and it is responsible for almost 4% of all deaths caused by cancer in men (5). Treatments with immune checkpoint inhibitors that have proved successful in a number of cancers have not shown the same responses for prostate cancer patients where only a minority of patients have seen benefits (6, 7). This has been shown to be due to the low mutational burden seen in the disease, resulting in a small number of neoantigens and limited T cell infiltration: Prostate cancer patients who respond to agents such as anti-PD1 have mutations in genomic instability or DNA mismatch repair genes that increase neoantigen expression (8, 9). Therefore, finding novel immunotherapeutic agents that can recruit and drive the activation and proliferation of NK and other effector cell populations within the cancer microenvironment without requiring the presence of neoantigens is of major interest. One such molecule is Interleukin-15 (IL-15), a cytokine that can expand and activate NK cells and CD8 T cells (10) and can also recruit these cells into tumors: This latter property has been shown when the tumors are genetically modified to express IL-15 (11, 12) and is thought to involve upregulation of chemokines CXCL9 and CXCL10 (that attract CXCR3 bearing effector cells) by IL-15 (13) in the tissue.

IL-15 is a 14 kDa protein that belongs to the α-helix bundle of cytokines: This cytokine is widely expressed by many cell types, such as monocytes, macrophages, dendritic cells, and epithelial cells, but only acts after binding to a heterotrimeric receptor composed by a beta and a γ subunit, which are shared with other cytokines, and one subunit specific for IL-15, IL-15Rα (14). After being recognized by the heterotrimeric receptor, IL-15 can act at many levels including aiding the survival, differentiation, and priming of Natural Killer (NK) cells (15–17). Due to its many immunological functions, IL-15 has been considered one of the most promising immunotherapeutic molecules over the past decade (18). It has been previously shown by our group that IL-15, unlike other therapeutic cytokines such as IL-2 and IL-12, can stimulate expansion and activity of CD8 T cells and NK cells *in vitro* when they are exposed to prostate cancer cells (19). Moreover, we have shown that IL-15 localized in tumor lesions leads to an approximate 50% tumor reduction and increases survival of mice with prostate tumors with no apparent toxicity (20).

Another pathway that has generated increased scientific interest in recent years is the immunological response triggered by the STING (Stimulator of Interferon Genes) pathway. This protein is localized in the endoplasmic reticulum and was first identified in 2008 as a major player in DNA-mediated innate immunity (21). Later in 2013 the pathway involving STING in the activation of the innate immune system was unveiled by Chen and co-workers (22, 23). The authors determined that the recognition of cytosolic DNA by the intermediate cyclic GMP–AMP synthase (cGAS) results in the activation of STING by cyclic dinucleotides (CDN) and the consequent expression of type 1 interferons, particularly IFN-β. Since CDNs act as agonists of STING, these have been considered good candidates as immunotherapeutic agents. In this study, we have used the chemically synthesized CDN 2´3´-c-di-AM(PS)2 (Rp,Rp) as this is an analogue of 3’3’-cyclic adenosine monophosphate (c-di-AMP), a second messenger molecule produced by bacteria that causes a potent immunological response in mammals (24). This chemical CDN is also an analog of ADU-S100, a STING agonist injected intratumorally in mice leading to tumor reduction by inducing T-cell mediated immunity (25).

In this study we sought to investigate whether a combination of IL-15 and ADU-S100 analog [2´3´-c-di-AM(PS)2 (Rp,Rp)] could lead to an increased therapeutic potential in targeting prostate cancer using in an *in-vitro* prostate cancer-lymphocyte co-culture model, and if so, which are the main immune cell populations involved in the eradication of the cancer cells. We hypothesized that IL-15 could induce tumor cell death by increasing the activation and proliferation of NK cells through upregulation of type II interferons (IFNs) i.e. IFNγ, while ADU-S100 analog could activate type I interferons i.e. IFNα/β on effector cells, as previously shown in T cells (26) hence combining the two agents could lead to an augmented effect. We therefore performed *in vitro* co-culture experiments of prostate cancer cells with non-adherent peripheral blood mononuclear cells (PBMCs), treated with either IL-15 alone or a combination of IL-15 and the ADU-S100 analog. We examined the viability of the cancer cells within the co-cultures, and assessed the expansion of NK and CD8 T cells well as a number of surface and activation markers for these cells.

## Methods

### Cell Lines

All the cell lines used in this study were obtained in the last 3 years. PC3 and WPMY-1 cell lines were obtained from ATCC (LGC Standards, Teddington, UK), while LNCaP and PNT2 were purchased from ECACC (Public Health of England, Salisbury, UK). LNCaP and PC3 are human metastatic prostate epithelial carcinomas. WPMY-1 and PNT2 originate from stromal non-tumorigenic and normal prostate epithelial cells respectively and were SV40-immortalized. The LNCaP, PC3, and PNT2 cell lines were cultured in RPMI 1640 medium (Sigma Aldrich, Poole, UK) supplemented with 2 mM glutamine, 1% antibiotic antimycotic solution, and 10% fetal bovine serum (ThermoFisher Scientific, Paisley, UK). WPMY-1 cells were cultured in Dulbecco’s modified Eagle’s medium (DMEM) medium (Sigma Aldrich, Poole, UK) supplemented as described previously. Cells were maintained in a humidified atmosphere with 5% CO_2_ at 37°C and were tested negative for mycoplasma infection using LookOut Mycoplasma PCR (Sigma-Aldrich).

### Isolation of Non-Adherent Peripheral Blood Mononuclear Cells

Peripheral blood mononuclear cells (PBMCs) were isolated from healthy donors using anonymized leukodepletion cones (Blood Transfusion Service, NHS Blood and Transplantation). Blood from the leukodepletion cones was diluted 1:5 in Hanks’ balanced salt solution (Sigma Aldrich, Poole, UK) to a final volume of approximately 50 ml. PBMCs were then isolated after centrifugation at 450x*g* for 30 min (without deceleration) on a Histopaque 1077 density gradient (Sigma Aldrich, Poole, UK). The density gradient was achieved by layering 25 ml of diluted blood onto 15 ml of Histopaque. Remaining red blood cells were lysed by incubation of isolated PBMCs with RBC lysis buffer (Thermofisher, Paisley, UK) for 5 min at 37°C. Non-adherent PBMCs were obtained after 3 h incubation of isolated PBMCs on a 175 cm^2^ culture flasks containing 3 × 10^6^ cells/ml in 40 ml RPMI complete medium. After 3 h, the non-adherent cells were harvested for further use.

### Isolation of Subpopulations From Non-Adherent PBMCs

CD56^+^CD3^−^ NK cells, CD19^+^ B cells, and CD8 T cells were isolated from non-adherent PBMCs by using isolation kits provided by Miltenyi Biotech (Woking, UK). Pure populations were obtained by following manufacturer’s instructions and purity of populations was confirmed by flow cytometry.

### Lymphocyte-Cancer Cell Co-Culture Experiments

Prostate cancer cells (LNCaP or PC3) or non-tumorigenic prostate cell lines (PNT2 or WPMY-1) were seeded at a density of 6 × 10^4^ cells/well in 24-well plates and incubated for 3 h at 37°C with 5% CO_2_ to allow adhesion of the cells. After this period, non-adherent PBMCs were added to each well to achieve a ratio of 1:4 cancer cells: non-adherent PBMCs. Co-cultures were incubated for 48 h in the presence of either IL-15 (Peprotech, London, UK), the STING agonist 2’3’-c-di-AM(PS)2 (Rp/Rp) (herein designated as ADU-S100 analog, from Invivogen, Toulouse, France), or a mixture of IL-15 with 2’3’-c-di-AM(PS)2 (Rp/Rp). Control experiments were also prepared in which IL-15 was replaced by PBS and 2’3’-c-di-AM(PS)2 (Rp/Rp) by a linear dinucleotide, 2’3’-cGAMP Control (herein referred to as Control) (Invivogen, Toulouse, France). The same concentration of IL-15 (2.5 ng/ml) was used in all experiments while three concentrations of 2’3’-c-di-AM(PS)2 (Rp/Rp) and 2’5’-GpAp were tested (0.05, 0.5, and 1 µg/ml). After 48 h, cells from each condition were collected for further analysis.

Co-cultures with isolated populations of B- and CD8 T lymphocytes and NK cells were carried out as described above but using a 1:1 ratio for effector cells:PC3 and 0.5:1 for effector cells:LNCaP. For co-cultures using PBMCs depleted of NK cells, the number of total cells used corresponded to the total number of non-adherent PBMCs minus 10% to account for the absence of NK cells which on average comprise 10% of PBMCs.

### Flow Cytometric Analysis

For the evaluation of tumor cell death, co-cultures from each well were collected and stained with anti-human CD45-FITC and Live/Dead Fixable Near-IR Dead Cell staining (Thermo Fisher Scientific, Paisley, UK) for the quantification of lymphocytes and living cells, respectively. To validate the percentage of tumor cell death, each condition examined was spiked with a known concentration of PE-labeled BD quantibrite™ beads (BD Biosciences, Wokingham, UK). Viability of the CD45+ lymphocytes, NK cells (CD56+CD3− cells), and CD8 T cells was also analyzed with the different treatments in the presence or absence of the cancer cells.

Cells were also stained with fluorophore conjugated anti-human CD3, -CD8, CD4 -CD56, -CD19, and CD80 to identify CD8 T cells, CD4 T cells, NK cells, B cells, and activated B cells respectively. Fluorophore conjugated CD11c was used to identify macrophages/dendritic cells (27). Fluorophore conjugated anti-NKG2D, -perforin, -CD107a, and CD69, were used to analyze activation of NK and T lymphocytes (see **Supplementary Table 1** for clone details). The phenotypes of the cells in the non-adherent PBMCs were evaluated at the start of the incubation period, before the cells were placed into the co-cultures, to determine numbers of B cells, and macrophage/dendritic cells at the start of the co-cultures to evaluate whether there was a loss of these myeloid cell types after 48 h co-cultures with tumor cells. CD3, CD8 CD4, and NK cells were also evaluated before addition to co-cultures to determine that the numbers of these cells were in line with previous studies on non-adherent PBMCs derived from normal healthy donors. All samples were acquired on FACS Canto II and data analysis was carried out using FlowJo version 10 (see **Supplementary Figure 1** for gating strategy). All conjugated antibodies used in this study were purchased from Biolegend (London, UK).

### Absolute Quantification of Cell Death

To validate tumor cell death quantified by flow cytometry, an absolute quantification of living cancer cells was carried out with labelled ultracompetent beads. Ultracompetent beads (UltraComp eBeads™, Thermo Fisher Scientific, Paisley, UK) were stained with an anti-human CD3 antibody conjugated with pacific blue fluorophore. Staining was carried out according to manufacturer’s manual. After counting the number of beads per ml of buffer, 50 μl of labeled beads was added to each sample.

### Enzyme-Linked Immunosorbent Assay (ELISA)

Supernatants were collected from non-adherent PBMCs and prostate cancer cells co-cultures after centrifugation at 400x*g* for 5 min. Interferon-alpha (IFN-α) Interferon beta (IFN-β) (R&D systems, Abingdon, UK) and Interferon-gamma (IFN-γ) (Biolegend, London, UK) levels in the supernatants were determined by ELISA kits according to manufacturer’s instructions.

### Immunoblotting Experiments

The expression levels of STING in non-adherent PBMCs or prostate cancer cells in the presence of either IL-15 (Peprotech, London, UK), the ADU-S100 analog 2’3’-c-di-AM(PS)2 (Rp/Rp) (ADU-S100 analog), and a mixture of ADU-S100 analog with IL-15 or PBS was evaluated. Cells were seeded in 24-well plates following the protocol described above. After 48 h, cells were collected by centrifugation, medium was removed, and cells were washed once with PBS. To obtain protein extracts, cells were resuspended in RIPA buffer containing protease inhibitor (Roche, Switzerland) and incubated on ice for 10 min with occasional agitation. Cell debris were removed by centrifugation at 14000x*g* for 15 min at 4°C. Cleared supernatants were transferred into clean tubes and protein concentration was estimated by Pierce™ BCA Protein Assay (Thermo Fisher Scientific, Paisley, UK). For each sample, 20 μg of total protein was loaded onto a 4–12% SDS-polyacrylamide gel. Separated proteins were transferred to a nitrocellulose membrane using an iBlot^®^ Dry Blotting System from Invitrogen (Thermo Fisher Scientific, Paisley, UK). Membrane was incubated with Tris-buffered saline containing 0.1% Tween-20 and 3% BSA for 1 h to decrease unspecific binding. To investigate two proteins of different sizes, the membrane was cut horizontally. The upper membrane stripe was incubated with a mouse monoclonal anti-α-tubulin antibody (1/1,000, R&D, Bio-Techne Ltd, Abingdon, UK) and the lower membrane stripe was incubated with anti-human 1,000-fold diluted STING/TMEM173 antibody (R&D, Bio-Techne Ltd, Abingdon, UK) overnight at 4°C. In both cases, a secondary antibody coupled to a horseradish peroxidase was used further chemiluminescence detection (ECL western blotting detection from GE Healthcare).

### Statistical Analysis

Data were analyzed using GraphPad Prism 8 (GraphPad Software, La Jolla, CA, USA). Statistical significance of differences was determined by one-way ANOVA with Dunnett’s multiple comparisons post-tests, with a 5% level of significance. Results are presented as mean ± standard error of the mean (SEM).

## Results

### Effect of IL-15 in Combination With ADU-S100 Analog on Tumor Cell Death

**Figure 1:**
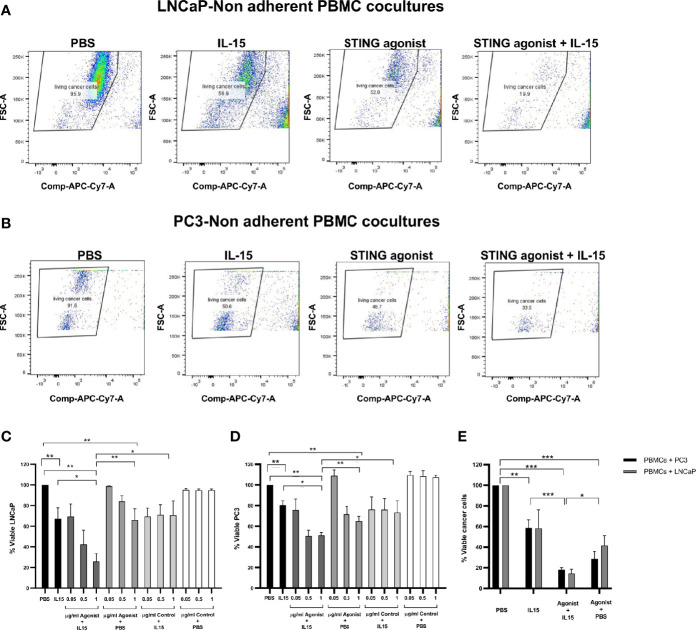
Viability of cancer cells after co-culture with non-adherent PBMCs for 48 h in the presence of IL-15 (2.5 ng/ml) or a mixture of IL-15 with different concentrations of the STING agonist 2’3’-c-di-AM(PS)2(Rp/Rp) (ADU-S100 analog) (designated as agonist). Panels **(A, B)** display representative FACs plots demonstrating the increased killing of LNCaP and PC3 cells respectively in co-cultures treated with STING agonist and IL-15 compared with IL-15 alone, STING agonist alone, or PBS. Panels **(C, D)** chart the viability of the LNCaP and PC3 cells respectively. Further controls were carried out by using a linear nucleotide (2’5’-GpAp) instead of the ADU-S100 (herein designated as Control). Panel **(E)** displays the absolute quantification of viable cells as determined using labeled ultracompetent beads. Results are means +/- SEM of triplicate or quadruplicate experiments (*p < 0.05, **p < 0.01, and ***p < 0.001, one-way ANOVA with Dunnett’s multiple comparisons post-test).

Tumor cell death was evaluated in co-cultures containing non-adherent PBMCs and either LNCaP or PC3 cancer cells incubated for 48 h in the presence of the cytokine IL-15, the ADU-S100 analog, or a combination of both. **Figure 1** summarizes the results obtained in each case. **Figures 1A**, **B** demonstrate a representative experiment displaying the killing of LNCAP cells and PC3 cells respectively as FACs plots and **Figures 1C, D** display the numerical values from multiple experiments carried out with LNCaP and PC3 cells respectively. When cancer cells were co-cultured with PBMCs in the presence of 2.5 ng/ml IL-15, there was a mean 33 and 22% of LNCaP and PC3 cell killing, respectively, compared to the control PBS. Also when incubated with 1 µg/ml ADU-S100 analog alone, there was a mean killing of 33 and 36% of LNCaP and PC3 cells compared to PBS alone: However, a significant increase in cell death (*p* < 0.05) compared to IL-15 alone or ADU-S100 alone was obtained when cancer cells were co-cultured with PBMCs in the presence of 2.5 ng/ml IL-15 in combination with 1 µg/ml ADU-S100 analog. Tumor cell death increased to 74 and 52% for LNCaP and PC3, respectively. When the ADU-S100 analog was replaced by the linear nucleotide 2’3’-cGAMP (herein named as Control), no cancer cell death was observed for both cell lines tested (**Figures 1C, D**, light gray and white bars) even at higher doses of the linear nucleotide in the absence of IL-15. An absolute quantification using labeled ultracompetent beads confirmed the results obtained (data shown in **Figure 1E**).

From visualizing our FACs data, we observed that the decrease in live tumor cells in the presence of the ADU-S100 and IL-15 combination was not due to a lack of proliferation of the tumor cells as we observed a clear reduction of live CD45 negative cells (**Figure 1** and **Supplementary Figure 1**) and an increase in dead cells.

To exclude the hypothesis of cancer cell death caused by drug toxicity, cancer cells were cultivated in the same conditions as described previously in the absence of non-adherent PBMCs. **Supplementary Figure 2** shows that for all the conditions examined, the viability of LNCaP after 48 h was 99 ± 2% (Panel A) and an identical result was obtained for PC3 cell line (100 ± 1.5%) (Panel B). The viability of the CD45+ population was also not significantly affected by the different treatments in the presence of LNCaP or PC3 cancer cells (**Supplementary Panels C, D** respectively) or in their absence (Panel E) although there was a small drop to 93% in the LNCaP-PBMC co-cultures and drop to 95% in the PC3-PBMC co-cultures with the 1 µg/ml ADU-S100 and IL-15 combination.

A 60% killing of the non-tumorigenic prostate stromal cell line WPMY-1 in the presence of either IL-15 or IL-15 in combination with ADU-S100 analog was also confirmed in the presence of non-adherent PBMCs, but the viability of the non-tumorigenic epithelial prostate PNT2 cell line was only affected by a maximal 30% by incubation with both IL-15 and ADU-S100 analog (see **Supplementary Figure 3**).

We considered other techniques to measure cell death (apart from live/dead staining) as there are numerous ways to detect dying cancer cells. We studied the possibility of using ATP, HMGB1, or calreticulin in addition to the live/dead staining we performed. However, our model is a co-culture of tumor cells with non-adherent PBMCs. Therefore, in measuring ATP, we would not be able to distinguish whether the dead cells were lymphocytes or tumor cells as ATP is released from any type of dying cell. HMGB1 is also secreted from both dying tumor cells and lymphocytes, but is also released when NK cells are activated (28). In the case of calreticulin, although this is a surface marker on dying tumor cells, it also comes to the surface in cytotoxic lymphocytes or NK cells when they release their granules to kill other cells (29). The technique of live/dead staining that we have chosen gives accurate, reproducible measurement of cell death and we have also confirmed the cytotoxicity using an absolute quantification of living cancer cells with labelled ultracompetent beads.

### Effect of Co-Cultures on NK, CD8 T Cell, and B-Lymphocyte Expansion and Activation

**Figure 2:**
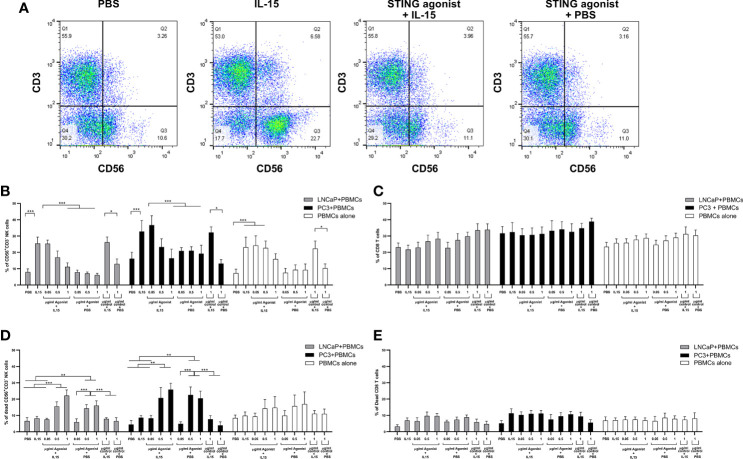
Expansion of NK and T cells from non-adherent PBMCs co-cultured with LNCaP or PC3 tumor cells for 48 h in the presence of IL-15 (2.5 ng/ml) or a mixture of IL-15 or PBS with different concentrations of the STING agonist 2’3’-c-di-AM(PS)2(Rp/Rp) (ADU-S100 analog- designated as agonist) or the linear nucleotide (2'5'GpAp) designated as control. Panel **(A)** displays representative FACs plots showing changes in CD56^+^CD3^−^ NK cell expression with the different treatments. Panel **(B)** displays percentages of CD56^+^CD3^−^ NK cells and panel **(C)** shows CD8^+^CD3^+^ T cells after co-culture with either LNCaP (gray) or PC3 (black). Controls were carried out by replacing IL-15 with PBS. Panels **(D, E)** show the percentage of dead CD56^+^CD3^−^ NK cells and CD8^+^CD3^+^ T cells respectively, with the different treatments. Results are means +/− SEM of triplicate experiments (*p < 0.05, **p < 0.01, and ***p < 0.001, one-way ANOVA with Dunnett’s multiple comparisons post-test).

We investigated the expansion of CD56^+^CD3^−^ NK cells and CD8 T cells in co-cultures as a mechanism for the increased cell death observed with the IL-15/ADU-S100 analog combination. **Figure 2A** shows representative FACs plots of CD56^+^CD3^−^ NK cells treated with PBS, IL-15, ADU-S100 analog, or a combination of IL-15 and ADU-S100 within co-cultures of lymphocytes and LNCaP cells. As expected, the presence of 2.5 ng/ml of IL-15 led to a significant increase (p < 0.001) in the number of NK cells when compared to the control PBS from a mean of 8% expression to 26% and from 16 to 38% for co-cultures containing LNCaP and PC3, respectively (**Figure 2B**). Surprisingly, when 1 µg/ml ADU-S100 analog was used alone (with PBS control) or with IL-15, the percentage of NK cells did not change significantly from the control PBS expansion (from 8 to 11% and 16 to 19% for co-cultures containing LNCaP and PC3, respectively). When IL-15 was used together with 1 µg/ml of the control agonist (the linear dinucleotide, 2’3’-cGAMP), there was a similar expansion of NK cells compared to IL-15 alone. Similarly, when cancer cells were not present, the lower percentage of NK cells in the combined treatments (2.5 ng/ml IL-15 with 1 µg/ml ADU-S100 analog) had no statistical significance when compared to the presence of PBS alone (**Figure 2B**). In the case of CD8 T cells, no significant differences were observed in numbers for all examined conditions in both co-cultures examined as well as PBMCs cultured in the absence of cancer cells (**Figure 2C**).

To determine the reason for the drop in NK cell numbers in the presence of the ADU-S100 alone and with the combined IL-15, we investigated the number of dead NK cells (**Figure 2D**), comparing this to CD8 T cell death (**Figure 2E**) with the different treatments. IL-15 had no significant effect on NK cell viability. However, we observed that in PBMC-cancer cell co-cultures, NK cells treated with the ADU-S100 analog, with or without IL-15, had an increased population of dead cells (up to 30% dead cells) in the presence of the 0.5 and 1 µg/ml doses. There was no significant change in dead NK cells in the presence of 1 µg/ml control agonist (linear dinucleotide, 2’3’-cGAMP) with or without the IL-15. In the PBMCs alone cultured with the different treatments, we observed no significant differences in NK cell death with the different treatments.

The numbers of dead CD8 T cells were similar across all treatments and co-cultures (5–10%). The CD8 T cell death in the PBMCs cultured without cancer cells was slightly less (around 7% on average) but this was not significantly different from cell death in the PBMC-cancer cell co-cultures.

Numbers of B cells as measured by the expression of CD19 were unaffected by any of the treatments in the co-cultures or individual cells (**Supplementary Figure 4**). Activation of these cells as measured with CD80 was minimal as less than 10% of the B cells expressed this marker (**Supplementary Figure 4B**). The marker CD11c was used to identify macrophage/dendritic cell types in the co-cultures: These cells are known to express the STING pathway, and agonists of this pathway such as CDNs can stimulate the production of type 1 interferons that can activate NK and T cells to induce tumor death (30). Numbers of B cells and CD11c positive cells did not change after the 48 h co-culture with any of the treatments compared to numbers seen before co-culture (**Supplementary Figures 4A, C**). The percentages of CD3, CD8, CD4, and NK cells before addition to co-cultures were in line with previous studies on non-adherent PBMCs derived from normal healthy donors (31) and CD3 and CD4 cell numbers, in addition to CD8 T cell numbers, did not change with the different treatments, or after co-culture, compared to levels before co-culture (data not shown).

Activation of NK cells and CD8 T cells was investigated next, as a mechanism for the increased tumor killing. Extracellular CD69 and intracellular activation markers (perforin) were examined by flow cytometry in NK cells and CD8 T cells. Perforin is responsible for pore formation in the plasma membrane of target cells while CD107a, also known as LAMP-1, is a marker for degranulation in NK and CD8 T cells. The presence of CD107a is required for efficient trafficking of perforin (32).

**Figure 3:**
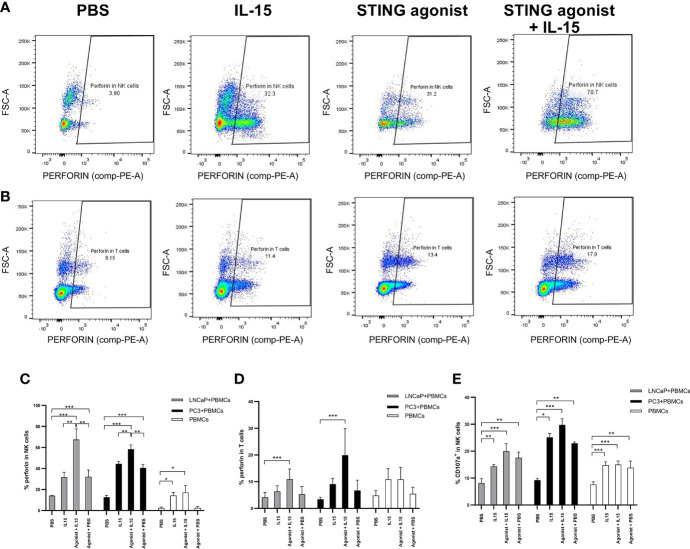
Activation of NK and T cells after co-culture of PC3 or LNCaP cells with non-adherent PBMCs for 48 h in the presence of IL-15 (2.5 ng/ml) or a mixture of IL-15 with different concentrations of the STING agonist 2’3’-c-di-AM(PS)2(Rp/Rp) (ADU-S100 analog- designated as Agonist). Panels **(A, B)** display representative FACs plots of perforin expression in gated CD56^+^CD3^−^ NK cells and in CD3^+^CD8^+^ T cells respectively, in non-adherent PBMCs co-cultured with LNCaP. Panels **(C, D)** display graphs showing the percentage expressions of perforin in NK cells (CD56^+^CD3^−^) and CD8^+^(CD3^+^) T cells respectively, co-cultured with LNCaP, PC3, or without cancer cells with the different treatments. Panel **(E)** shows the percentage of CD107a^+^ in CD56^+^CD3^-^ NK cells in co-culture with LNCaP (light gray) or PC3 (black). Results are means +/− SEMs of triplicate experiments (*p < 0.05 and ***p < 0.001, one-way ANOVA with Dunnett’s multiple comparisons post-test).

**Figures 3A, B** display representative FACs plots showing perforin expression by NK cells and CD8 T cells in co-cultures incubated with the different treatments. An increased NK cell perforin expression was observed in co-cultures of PBMCs with LNCaP or PC3 cells treated with ADU-S100 and IL-15 (mean of 67 and 58% respectively) compared with IL-15 alone (mean of 31 and 44% for LNCaP and PC3 co-cultures respectively), agonist alone (means of 32 and 41% for LNCaP and PC3 respectively) or PBS (means of 2.4 and 2.2% for LNCaP and PC3 respectively) (**Figure 3C**). In the absence of cancer cells, there was a similar, smaller increase of perforin expression with IL-15 and ADU-S100 (17%) and IL-15 alone (14%) compared to the PBS control (2.2%). The same trend was seen with CD8 T cells (**Figure 3D**) with the greatest increases in perforin expression occurring in co-cultures treated with the agonist and IL-15 combination, although the maximal perforin expression occurring with IL-15 and ADU-S100 was lower in LNCaP and PC3 co-cultures respectively (10 and 17%).

The expression of CD107a by NK cells also increased when cells were treated with either IL-15 or IL-15 combined with ADU-S100 analog (**Figure 3E**), and was also higher in PBMC-cancer cell co-cultures compared with PBMCs incubated without cancer cells, however, there was no significant differences in expression with IL-15 compared to the IL-15 and ADU-S100 combination. CD107a was observed only in very small levels on CD8 T cells (data not shown) as previously reported (33).

**Figure 4:**
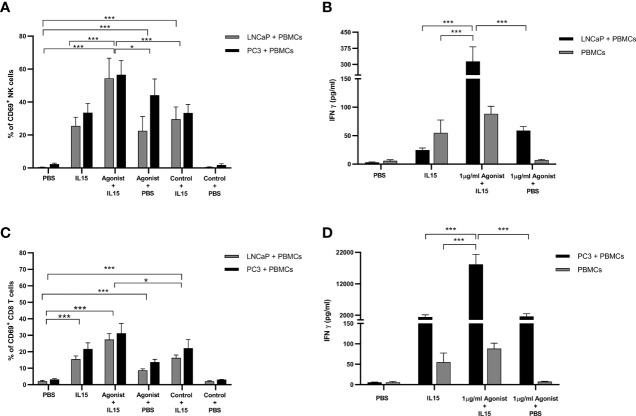
Expression of CD69 by NK and T cells and secretion of Interferon gamma (IFN-γ) after co-culture of PC3 or LNCaP cells with non-adherent PBMCs for 48 h in the presence of IL-15 (2.5 ng/ml) or a mixture of IL-15 with different concentrations of the STING agonist 2’3’-c-di-AM(PS)2(Rp/Rp) (ADU-S100 analog). **(A, C)** Percentage of CD69^+^ in CD56^+^CD3^−^ NK cells and CD8^+^CD3^+^ T cells, respectively **(B, D)** concentration of extracellular interferon γ (IFN-γ) after 48 h in co-culture with either LNCaP (gray) or PC3 (black). Controls were carried out by replacing IL-15 with PBS or replacing the ADU-S100 analog agonist with the linear nucleotide (2’5’-GpAp -designated as Control) . Results are means +/− SEM of triplicate experiments (*p < 0.05 and ***p < 0.001, using one-way ANOVA with Dunnett’s multiple comparisons post-test).

One of the earliest cell surface markers expressed by NK cells and CD8 T cells after activation is CD69 (34). Therefore, this protein is a good candidate to examine activation of NK and CD8 T cells in our study. As expected, only a small percentage (1.5 to 3%) of NK and CD8 T cells expressed CD69 in the presence of PBS (**Figures 4A, C**). When PBS was replaced by treatments, an increase in the expression of CD69 was observed. Again, NK cells co-cultured with cancer cells in the presence IL-15 combined with ADU-S100 analog expressed the highest amounts of CD69 (a mean of 54 and 57% respectively for LNCaP and PC3 co-cultures respectively, compared to a mean of 25 and 33% expression for LNCaP and PC3 co-cultures respectively, with IL-15 alone and 23 and 44% for LNCaP and PC3 co-cultures respectively, with ADU S-100 analog alone (**Figure 4A**). In CD8 T cells, the pattern of increased CD69 expression was similar with the highest expression seen with the combined treatments, but again with a smaller increase seen in the IL-15/ADU-S100 analog combination (a mean of 27 and 32% respectively in LNCaP and PC3 co-cultures (**Figure 4C**).

This observation, together with the presence of perforin and CD107a, shows that the presence of cancer cells and both treatments led to a higher activation of NK cells. The activatory receptor NKG2D, expressed on a number of cytotoxic effector cell types, was upregulated by IL-15, as we have previously observed (19), and by the ADU-S100 analog, but there was no additive expression in the presence of both agents (**Supplementary Figure 4**).

### Production of IFNγ, but Not IFNα or IFNβ by Lymphocytes Co-Cultured With Cancer Cells

It has been reported in the literature that activation of STING by cyclic dinucleotides triggers the production of type I interferons (IFN-α/β) (21), while stimulation of lymphocytes with IL-15 leads to the production of interferon gamma (IFN-γ) (35). Therefore, the presence of IFN-α, IFN-β, and IFN-γ in supernatants of PBMCs co-cultured with cancer cells was investigated by ELISA. Surprisingly, levels of IFNα and IFN-β were minimal for all conditions examined with concentrations around 10 pg/ml (data not shown). As expected, in the presence of IL-15, levels of IFN-γ increased by 8-fold in co-cultures with LNCaP and 250-fold in co-cultures with PC3, from 6 pg/ml to 1,411 pg/ml (**Figures 4B, D**). Surprisingly, when 1 μg/ml ADU-S100 analog was added to IL-15, the accumulation of IFN-γ increased again by 12- to 13-fold change for both co-cultures examined. Although the fold-change was identical in both co-cultures, it is important to note that, in the presence of PC3 cells, concentrations of IFN-γ are much higher (18,220 pg/ml *versus* 313 pg/ml for PC3 and LNCaP respectively). Cultivation of PBMCs alone in the presence of IL-15 or combined with ADU-S100 analog produced smaller amounts of IFN-γ (up to 100 pg/ml) (**Figures 4B, D**).

### Expression of STING by PBMCs and Cancer Cells

**Figure 5:**
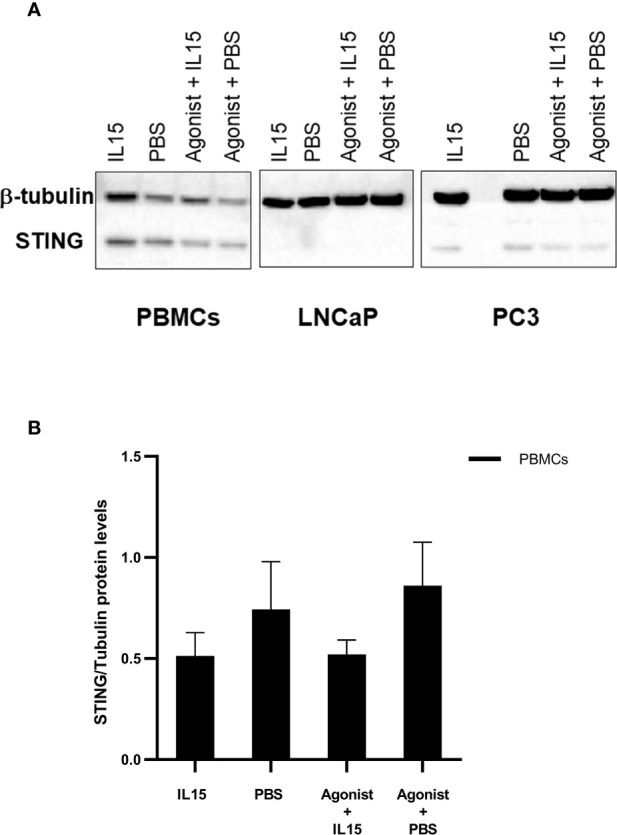
Changes in the expression levels of STING in PBMCs, LNCaPs, and PC3s when incubated with PBS, IL-15, STING agonist, or a combination of both. Semi-quantitative western blot using anti-STING (40 kDa) and anti-β-tubulin (52 kDa) antibodies on protein extracts from PBMCs, LNCaP, and PC3 cells. Cells were incubated for 48 h with either IL-15 (2.5 ng/ml) or a mixture of IL-15 with 1 μg/ml of the ADU-S100 agonist analog 2’3’-c-di-AM(PS)2(Rp/Rp- designated as Agonist). Controls were carried out where IL-15 was replaced by PBS. Representative blots of three biological replicates are shown in the upper panel **(A)** to demonstrate that protein bands of the expected molecular weights were detected. The lower panel **(B)** corresponds to densitometric analysis of protein amounts for PBMCs.

To evaluate the expression patterns of STING in PBMCs, LNCaP, and PC3, cells were cultured as described previously in the presence of PBS, IL-15, ADU-S100 analog or the combination of IL-15 with the ADU-S100 analog. After 48 h cells were collected, and the expression of STING was evaluated by western blotting. Interestingly, despite the minimal levels of expression, presence of STING protein was confirmed in both LNCaP and PC3 cells (**Figure 5A**). Expression levels of STING protein were higher in PBMCs, but no significant changes were observed for all the conditions tested (**Figure 5B**).

### Effects of Lymphocytes Pre-Incubated With ADU-S100 Analog in the Killing of Cancer Cells

**Figure 6:**
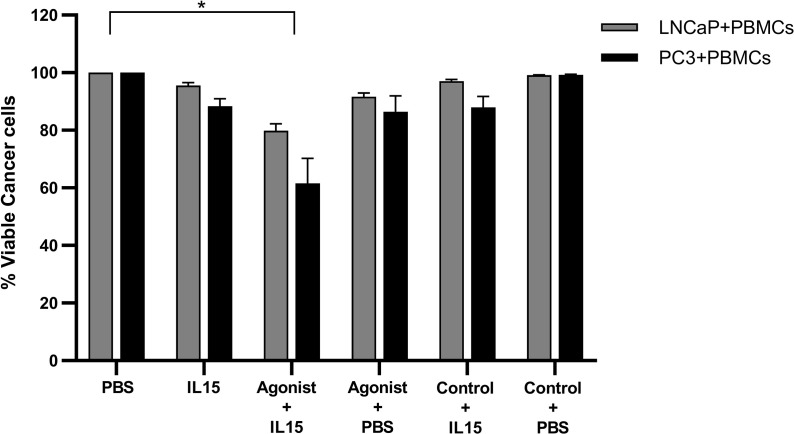
Viability of cancer cells after co-culture with pre-activated non-adherent PBMCs. Percentage of viable PC3 (Black) and LNCaP (gray) after being co-cultured for 48 h with PBMCs which were previously incubated for 24 h with IL-15 (2.5 ng/ml) or a mixture of IL-15 with 1 μg/ml of the STING agonist 2’3’-c-di-AM(PS)2(Rp/Rp) (ADU-S100 analog, termed agonist). Controls were carried out by replacing IL-15 with PBS and the agonist with a linear nucleotide (2’5’-GpAp), herein designated as Control. Results are means +1 SEM of triplicate (*p < 0.05 compared to PBS control, by one-way ANOVA with Dunnett’s multiple comparisons post-test).

To understand if the effect observed previously in cancer cell death was solely mediated *via* non adherent PBMCs, or through the cancer cells, the former were preincubated with IL-15, ADU-S100 analog, or a combination of both for 24 h under optimal growth conditions and then washed before addition to the cancer cells for 48 h. Cancer cell death in the presence of either IL-15 alone or IL-15 in combination with ADU-S100 analog was observed (**Figure 6**), but to a less extent than described above (**Figure 1**): IL-15-pre-incubated PBMCs resulted in 5 and 12% of LNCaP and PC3 cell death, respectively. When pre-activation was carried out with the combination of IL-15 and the cyclic nucleotide, cell death of LNCaP and PC3 increased to 20 and 39%, respectively (**Figure 6**).

### Effect of Purified NK, CD8, and B Cell Populations in the Killing of Cancer Cells

**Figure 7:**
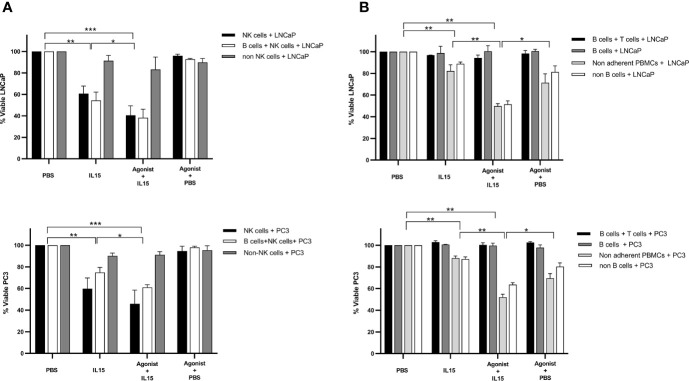
Viability of cancer cells after co-culture with purified populations of lymphocytes for 48 h in the presence of IL-15 (2.5 ng/ml) or a mixture of IL-15 with different concentrations of the STING agonist 2’3’-c-di-AM(PS)2(Rp/Rp) (ADU-S100 analog- designated as Agonist). Effect of **(A)** pure NK cells, NK cells plus B cells, and non-adherent PBMCs depleted of NK cells and **(B)** pure B plus T cells, B cells alone, and non-adherent PBMCs depleted of B cells, compared with non-adherent PBMCs on the killing of cancer cells. Controls were carried out by replacing IL-15 with PBS. Results are means +/− SEM of quadruplicate experiments. (*p < 0.05, **p < 0.01, and ***p < 0.001 by one-way ANOVA with Dunnett’s multiple comparisons post-test).

Most results obtained so far led to the hypothesis that NK cells have a major role in the killing of cancer cells in our co-culture model. Therefore, to validate this hypothesis pure populations of NK, B, and CD8 T cells were isolated from non-adherent PBMCs using kits provided by Miltenyi. Purity of isolated populations was measured to be of approximately 95% for all cases (data not shown). No killing was observed for all conditions examined for B cells and T cells co-cultured with cancer cells (**Figure 7**). B cells alone also did not cause any significant killing of tumor cells, and depletion of B cells from non-adherent PBMCs did not significantly diminish the killing seen with the non-adherent PBMCs. The only pure population of cells that mediated killing were the NK cells (**Figure 7A**). The levels of NK cell mediated killing observed with IL-15 alone, and the combination of IL-15 and STING agonist were similar to that seen with the non-adherent PBMCs (a mean of 40 and 60% killing of LNCaP respectively, and a mean of 46 and 60% respectively with PC3). However, there was no killing effect seen with NK cell-tumor cell co-cultures treated with STING agonist alone. B cells added to the NK cells did not add significantly to the tumor cell death with the different treatments (**Figure 7A**). When NK cells were removed from the non-adherent PBMC population, there was no significant tumor cell death with any of the treatments.

## Discussion

The current study was conducted to evaluate the effect of IL-15 in combination with the ADU-S100 analog 2’3’-c-di-AM(PS)2 (Rp,Rp) as immunotherapeutic agents for the treatment of prostate cancer. To our knowledge, this is the first study published so far evaluating the benefits of ADU-S100 analog in combination with IL-15 in the treatment of solid tumor models. In addition to IL-15, which has been considered a promising immunotherapeutic agent to treat prostate cancer due to its ability to activate immune cells, in particular NK cells (19), the ADU-S100 analog 2’3’-c-di-AM(PS)2 (Rp,Rp) was also chosen due to its ability to stimulate type I interferon (IFN) expression in immune cells and convert cold tumors into immunologically hot tumors in some models of solid tumors (36, 37). Experiments were performed *in vitro* using peripheral blood mononuclear cells (PBMCs) from healthy donors and model prostate cancer cells (LNCaPs and PC3) as well as non-tumorigenic cell lines, PNT2 and WYPM1. Our results show that the combination of IL-15 with higher doses of the ADU-S100 analog led to a significant increase in cancer cell death when compared to the presence of IL-15 or ADU-S100 alone or PBS with PC3 and LNCaP cell lines. This increase was more drastic in the case of LNCaP cell lines, with only approximately 30% of the cancer cells remaining viable after exposure to the combination of treatments for a period of 48 h, whereas with PNT2, only a maximal 30% killing was observed with the combination. In the non-tumorigenic cell lines, cell death occurring with the IL-15 + ADU-S100 analog combination was not significantly greater than with IL-15 or ADU-S100 analog alone. It is important to note that no direct toxicity on cancer cells was observed for all the examined doses of ADU-S100 analog. This observation is in agreement with the results published by Ghaffari et al. (38), where 0.1 to 100 µg/ml of ADU-S100 analog was incubated *in vitro* for 48 h with the high-grade serous carcinoma (HGSC) cells and no toxicity was observed. Therefore, killing of cancer cells in the co-cultures in our study is mediated by the cell populations within the non-adherent PBMCs, rather than direct STING agonist toxicity. The ADU-S100 analog does however affect the viability of NK cells within the co-cultures, although not when non-adherent PBMCs were cultured alone.

To investigate the mechanisms of the increased cancer cell death, we first evaluated the expansion of NK cells in the lymphocyte: cancer cell co-cultures: We observed that the percentage of NK cells did not significantly change from the PBS control when the combination of IL-15 and ADU-S100 analog, or the ADU-S100 analog alone was used, although significant expansion was seen with IL-15 alone as previously described by ourselves and others (19, 39). In addition, a recent *in vivo* study has shown an increase in the proliferation and activation of NK cells when cyclic dinucleotides are present in the milieu of the cells (40)—this is not in agreement with our study where NK cell numbers went down in the presence of the ADU-S100 analog agonist, but not with its linear dinucleotide, 2’3’-cGAMP control. When we investigated NK cell death as a reason for the decreased numbers of NK cells with the ADU-S100 agonist with or without IL-15, we observed a marked increase in dead cells (up to 30%) in all treatments with the agonist at 0.5 µg/ml and 1 µg/ml. The numbers of dead cytotoxic CD8 T cell numbers however remained constant for all conditions examined, concomitant with a similar level of CD8 T cell numbers observed among all treatments. When non-adherent PBMCs were treated with the different agents alone, in the absence of tumor cells, viability of the NK cells was not significantly affected by the ADU-S100 analog.

As NK cell numbers were reduced in the ADU-S100 combination compared to IL-15, despite a marked decrease in tumor cell viability in the presence of the combination treatment, we investigated the activation of NK cells and CD8 T cells with the different treatments as a possible reason for the increase in tumor cell killing. Levels of perforin, and CD69 were all increased in NK and CD8 T cells when cells were treated with a combination of IL-15 and ADU-S100 analog in co-cultures compared with PBS, agonist, or IL-15 alone. CD107a was however, similarly increased by IL-15 alone and in combination with ADU-S100 analog in NK cells. Although a significant increase in activation markers was observed for both types of immune cells, levels of perforin and CD69 were much higher in NK cells than CD8 T cells (**Figures 3C, D** and **4A, C**) with the combination treatments.

In our previous publication we also observed upregulated expression of NK cell perforin and NKG2D with IL-15, that increased in the presence of tumor cells (19). We had previously considered whether this effect is dependent on NK cell contact with tumor cells and showed that this was not dependent on lymphocyte-tumor cell contact as similar activation was observed when we used a 0.4 micron filter between the lymphocytes and tumor cells (unpublished data). Based on our previous study, we would suggest that the upregulation of perforin in the NK cells here is also not due to cell-cell contact between tumor cells and NK cells, although it would be interesting to confirm this using our filter approach. However, it is known that perforin release itself is a contact-dependent phenomenon (41).

CD69 has also been shown to be upregulated only when NK cells are cocultured with tumor cells (42), and therefore we did not measure this marker in PBMCs alone. The increased expression of CD69 on NK cells matched the increase in dead NK cells in the co-cultures. Therefore, we speculated that the reason for the greater number of dead NK cells after stimulation with IL-15 and ADU-S100 analog may have been possibly caused by an over-activation of these cells. Although we did not measure the well known exhaustion markers PD-1, CD95, and TIM-3 (43), there is recent evidence that CD69 may act as marker for lymphocyte exhaustion and is an immunoregulatory marker in addition to being an early activation marker (44): A study in mice showed that T cell infiltration and killing of tumor cells was upregulated in the presence of antibodies to CD69 and also in CD69 knockout mice.

It is also known that STING activation results in cell apoptosis. For instance, STING agonists cause apoptosis of immune cells, including B cells and T cells, *in vitro* and *in vivo* (45) and so the reduction in NK cell numbers may be also be due to this in addition to, or alternatively to an overactivation of the NK cells.

As expected, an increase in the levels of IFN-γ was observed when PBMCs alone or co-cultured with prostate cancer cells were incubated with IL-15 (**Figure 4B**). T cells, NK cells, NKT cells, dendritic cells, and B cells have been described as the main producers of IFN-γ in the immune system after activation with interleukin-2 (IL-2) and IL-15 (46). Surprisingly, levels of IFN-γ increased to a much greater extent in the presence of IL-15 and ADU-S100 analog, compared to IL-15 alone in co-cultures with LNCaP and PC3, suggesting that IFN-γ activation of effector killer cells correlates to, and possibly could be mediating the increased killing. To identify the effector cells that are secreting the IFN-γ, we have previously studied intracellular cytokine expression of individual cell types using flow cytometry (data not shown) but have found that the levels detected are very low (<5% expression) in our model, in the absence of PMA/Ionomycin stimulation (the latter can mask the effects of homeostatic cytokines such as IL-15 or IL-2). We can infer from the greater levels of CD69 expression and perforin expression on NK cells, that it is likely that the majority of the interferon γ may be being secreted by the NK cells. This needs to be investigated further in future studies. Interestingly, an increase in IFN-γ caused by the presence of STING has also been reported (38) *in-vivo* where Ghaffari and co-authors reported an increase in tumor cell death caused by high levels of IFN-γ that induce the expression of immunosuppressive agents, such as PD-L1. We would therefore predict a potent anti-tumor response *in-vivo* with the IL-15/ADU-S100 combination. We did not however, see any secretion of IFNα or β unlike previous studies by Ishikawa and colleagues (21, 36), possibly as they had focused on STING activation of macrophages and dendritic cells that are the cell types that predominantly produce the type 1 interferons. Larkin and co-authors then demonstrated the direct role of STING in the activation of T cells followed by the production of IFN-beta (26), however, they were using murine T cells and a different agonist (DMXAA) which is not active on human STING receptors.

In this study, the population of immune cells utilized were non-adherent lymphocytes to reduce/eliminate the presence of macrophages and dendritic cells as we wanted to investigate a direct effect of STING agonists on T cells and NK cells. The presence of very minor numbers of macrophages or DCs in our lymphocyte populations was further validated by flow cytometry using the surface marker CD11c (**Supplementary Figure 4**). Phenotyping of the non-adherent PBMCs before the start of the co-culture and after the 2-day co-culture showed that CD11c levels comprised 1% of the population, and the levels of B cells also did not change significantly (comparing **Supplementary Figure 4C** with **Supplementary Figure 4A**). Average numbers of CD3, CD4, and CD8 expressing cells were not significantly different before or after 48 h co-culture. NK cell levels only changed upon treatment with the IL-15 (**Figure 2A**). The monocyte population defined by CD14 in non-adherent PBMCs has previously been measured by us (unpublished data) and others (47), demonstrating that CD14 cells make up less than 2% of the non-adherent PBMC population. It is thus improbable (but not impossible) that myeloid cells are contributing to the augmented killing caused by the two agent combination. Since we did not observe an increase in the number of CD8 T cells, and the production of perforin and expression of CD69 was most apparent in NK cells, and not CD8 T cells with the combination treatment, we hypothesize that the presence of IL-15 and ADU-S100 analog may cause an additive or synergistic effect on NK cells leading to an increase in the levels of IFN-γ.

We also confirmed that depletion of NK cells from the co-cultures resulted in a lack of killing with any agent or combination (**Figure 7**). This suggests that NK cells are critical for the killing effect as previously observed with IL-15 (19). When purified NK cells were used in the co-cultures, killing was observed with IL-15 alone, and the combination treatment. However, as there was no significant killing when NK cell-tumor cell co-cultures were treated with STING agonist alone, factors from other cell types are required to mediate killing in the presence of STING agonist. The killing of the prostate cancer cells was not, however, due to CD8 or B cells in the co-cultures: neither purified B cells, or B plus T cells, mediated any tumor cell killing. B cell depletion did not diminish the killing of PC3 or LNCaP cells in the co-cultures when compared to the levels seen with the non-adherent PBMCs. B cells were also not activated by any of the treatments as shown by the low levels of CD80 expressed on them with all the treatments (**Supplementary Figure 4**).

The requirement of cancer cells in the additive killing effects of ADU-S100 analog/IL-15 was demonstrated as we saw a diminished killing when STING and IL-15 was preincubated with PBMCs alone before the 48 h co-culture (**Figure 6**). However the additive effect of the IL-15 and ADU-S100 analog was still observed in these co-cultures and therefore STING in the non-adherent PBMCs is important for this effect.

A number of publications, (including those in which STING has been overexpressed in cells) has demonstrated that the levels of expression of STING correlate with its subsequent activity in a number of cell types (48–50). Therefore, we posited that the observation of an increased STING expression in lymphocytes or cancer cells may correlate with the increase in STING activity, leading to NK cell and T cell activation. The presence of STING protein in both cancer cell lines and non-adherent PBMCs was confirmed by immunoblotting. However, STING protein levels were very low in LNCaP and PC3 when compared to non-adherent PBMCs. Therefore, ADU-S100 analog is most likely acting mainly *via* STING in immune cells. The protein levels of STING in non-adherent PBMCs did not change significantly with the different treatments. Currently, homeostatic regulation of STING is still poorly understood, although it is known that presence of STING is finely regulated and it has been suggested that the balance between stabilization and turnover of STING most likely differs for different immune cell types (51). Either regulatory mechanisms keep STING protein levels stable under different conditions or excess of STING had been already degraded since protein levels were evaluated 48 h after incubation with ADU-S100 analog.

The phosphorylation of TBK1 and IRF3 has been studied as measures of STING activation (52), however, we did not measure this for two reasons. Firstly, STING expression is required for TBK1 activation, however, the NF kappa B pathway (that leads to increases in cytokine expression) can be stimulated by STING without TBK1 involvement (53). Secondly, IRF3 may also be activated in the absence of STING expression through the TLR pathway (54): IRF3 may also be activated by STING through the IF116 molecule or through the RIG-I-like receptors (RLRs) which also does not require TBK1.

The lack of Interferon type 1 production in our PBMC cocultures may also be explained by the possibility that STING in our cells was being activated by the ADU-S100 analog through the IF116 molecule, which would not require the expression of TBK1, and hence the TBK1 activation of IRF3 (which leads to production of type I interferons) may not occur. Indeed, TBK1 is not active in wild type T cells in mice or in Jurkats, unless the cells are stimulated by PMA/Ionomycin (55). Others have also shown that STING activation of NK cells does not result in the production of IFNβ in these cells (56). IRF3 activation (possibly through IF116 or RLRs) does however stimulate IFN-γ production in lymphocytes (57, 58), in line with our findings.

The mechanisms by which the ADU-S100 analogue and IL-15 combination affects the NK mediated killing of the cancer cells is not yet clear. However, It is known that perforin and IFN γ upregulated in the co-cultures treated with the combination are both capable of directly killing tumor cells: Perforin at the lytic synapse between NK cells and tumor cells induces pore formation in the latter, followed by a release of granzymes A and B which are packaged with perforin (59). The released granzymes then can induce cell apoptosis either through the activation of caspases (granzyme B) or through caspase independent mitochondrial membrane disruption (granzyme A) (60). IFN-γ can cause tumor cell apoptosis by increasing caspase-1, -3, -8 expression in the cells: It also upregulates secretion of FAS and FAS ligand and TNF-related apoptosis-inducing ligand (TRAIL) (61). IFN-γ can also induce a form of necrotic death, known as necroptosis, that involves serine–threonine kinase RIP1 (62). However, in most cancer cells, including prostate cancer cell lines PC3 and LNCaP, RIP1 is suppressed by caspase 8 (63) (64). Therefore it is more likely that the increased cell death we are seeing is through apoptosis mediated by both upregulated IFN-γ and perforin.

Another mechanism by which cell death has been shown to occur with NK cell mediated cytotoxicity toward tumor cells is through antibody dependent cell cytotoxicity (ADCC) (65). We do not think that this was a contributing mechanism to killing in our model due to the lack of killing seen with B cell – tumor cell co-cultures and the lack of suppressed killing when B cells were depleted from the co-cultures.

Further studies are required to dissect the mechanisms of cell death and to understand whether the combination of agents are also inducing necrotic death, and whether the apoptosis caused by the perforin and IFNγ results in immunogenic death.

*In vivo* studies using mice models are currently ongoing to further validate the effect of the combined treatment of IL-15 with ADU-S100 analog in the reduction of prostate cancer tumors. If tumor growth is inhibited, tumors and their infiltrating leukocytes will be analyzed in depth to determine changes in gene expression, where possible at the single cell level. The effects on tumor cell killing that we have observed in a simplified *in-vitro* co-culture system could be radically enhanced in the *in-vivo* environment. Additional presence of dendritic cells, macrophages, and other antigen presenting cells in the *in-vivo* tumor lesion milieu should give additional type I interferon activity which leads to activation of NK cells and CD8 Tcells (66). IFNγ is known to increase the recruitment of NK and CD8 T cells into the tumor microenvironment by its upregulation of the chemokines CXCL9 and CXCL10 (61)—a property that is shared with IL-15 (13). IFN-γ can also inhibit angiogenesis by mediating death of blood vessel endothelial cells in the tumor through their IFN-γ receptors (67).

To conclude, our study shows that the combination of IL-15 and ADU-S100 analog induces an activation of NK cells, and a resulting prostate cancer cell death much greater than either two agents used separately. If this marked tumor cell death is confirmed *in vivo*, this two agent combination may be a major advance in the treatment of Prostate cancer.

## Data Availability Statement

The original contributions presented in the study are included in the article/**Supplementary Material**. Further inquiries can be directed to the corresponding authors.

## Author Contributions

AE designed and performed experiments, analyzed and interpreted data and wrote manuscript. EP designed experiments, helped with acquiring funding, acquired and advised on STING agonists, and reviewed and critiqued data and the manuscript. PD obtained funding and contributed to concept design. CG obtained funding, created study, designed experiments, analyzed data, reviewed and revised/amended manuscript. All authors contributed to the article and approved the submitted version.

## Funding

The authors are grateful for the financial support from the charity Prostate Cancer Research (grant 6938). We would also like to acknowledge pilot funding support from the Cancer Research UK Kings Health Partners Centre at Kings College London.

## Conflict of Interest

The authors declare that the research was conducted in the absence of any commercial or financial relationships that could be construed as a potential conflict of interest.

## Supplementary Material

Supplementary Figure 1 Example of gating strategy applied in this study using FlowJo (V10). **(A)** Example of gating for the selection of living cancer cells (represented with LNCaP) and living CD45+ cells (non-adherent PBMCs) with plots shown of treatment with PBS, 2.5 ng/ml IL-15, 1 µg/ml STING agonist + 2.5 ng/ml IL-15, and 1 µg/ml STING agonist + PBS. **(B)** Gating strategy followed for the selection of viable NK and T cells. **(C)** Analysis of perforin expression in viable NK and CD8 T cell populations after 48 h incubation, showing the plots for PBS treatment.

Supplementary Figure 2 Viability of cancer cells without the presence of non-adherent PBMCs, and non-adherent PBMCs with and without the presence of cancer cells after incubation for 48 h in the presence of IL-15 (2.5 ng/ml) or a mixture of IL-15 with different concentrations of the STING agonist 2’3’-c-di-AM(PS)2(Rp/Rp) (ADU-S100 analog- designated as Agonist). Panel **(A)** shows LNCaP and Panel **(B)** shows PC3 cancer cells treated with the agents in the absence of PBMCs. Panel **(C)** and panel **(D)** show viability of CD45+ cells (non-adherent PBMCs) in the presence of LNCaP and PC3 cells respectively. Panel **(E)** shows viability of CD45+ cells cultured in the absence of cancer cells. Controls were carried out by replacing IL-15 with PBS and the agonist with a linear nucleotide (2’5’-GpAp), herein designated as Control. Results are means +/− SEM of triplicate or quadruplicate experiments.

Supplementary Figure 3 Viability of non-tumorigenic prostate cells after co-culture with non-adherent PBMCs for 48 h in the presence of IL-15 (2.5 ng/ml) or a mixture of IL-15 with different concentrations of the STING agonist 2’3’-c-di-AM(PS)2(Rp/Rp) (ADU-S100 analog- designated as Agonist) or the linear nucleotide (2’5’-GpAp -designated as Control). **(A)** WPMY-1 and **(B)** PNT2 prostate cell lines. Results are means +/− SEM of triplicate or quadruplicate experiments (*p < 0.05, **p < 0.01 and ***p < 0.001 by one-way ANOVA with Dunnett’s multiple comparisons post-test).

Supplementary Figure 4 Expansion of B cells and dendritic cells and the expression of activation markers CD80 on B cells, and NKG2D on NK cells or CD8 T cells after co-culture of PC3 or LNCaP cells with non-adherent PBMCs for 48 h in the presence of IL-15 (2.5 ng/ml) or a mixture of IL-15 with different concentrations of the STING agonist 2’3’-c-di-AM(PS)2(Rp/Rp) (ADU-S100 analog). Panel **(A)** displays the percentage expression of CD19 or CD11c as markers for B cells and macrophage/dendritic cells respectively in populations of non-adherent PBMCs co-cultured with PC3 or LNCaP cells. Panel **(B)** displays the expression of CD80 on B cells. In these experiments, cells were incubated for 48 h with either IL-15 (2.5 ng/ml) or a mixture of IL-15 with 1 μg/ml of the ADU-S100 agonist analog (2’3’-c-di-AM(PS)2Rp/Rp-designated Agonist). Controls were carried out where IL-15 was replaced by PBS or where ADU-S100 was replaced by the linear nucleotide (2’5’-GpAp-designated as Control). Panel **(C)** displays phenotypes of the cell populations in the non-adherent PBMCs at the start of the experiment, and confirms that the percentages of B cells and macrophage/DC populations (as measured with the CD11c marker) before non-adherent PBMCs were placed in the co-cultures, were unchanged from the levels seen after 48 h. Numbers of CD3, CD8, and CD4 T cells seen confirmed typical numbers previously observed in non-adherent PBMCs (31). Panels **(D)** and **(E)** show the percentage expression of NKG2D receptors on NK cells or CD8 T cells respectively when incubated with either IL-15 (2.5 ng/ml), ADU-S100 analog, or a mixture of IL-15 with 1 μg/ml of the ADU-S100 agonist analog 2’3’-c-di-AM(PS)2(Rp/Rp) with PBS as a control. Results are expressed as means +/− SEM of triplicate or quadruplicate experiments (*p < 0.05 and **p < 0.01, by one-way ANOVA with Dunnett’s multiple comparisons post-test).

Supplementary Table 1 Antibodies and fluorophores used on this study for flow cytometry.
