# Supplementary figures and images for: Combination of Interleukin-15 With a STING Agonist, ADU-S100 Analog: A Potential Immunotherapy for Prostate Cancer
Source: Front Oncol. 2021 Mar 10;11:621550. doi: 10.3389/fonc.2021.621550 (PMC7988118; doi:10.3389/fonc.2021.621550)

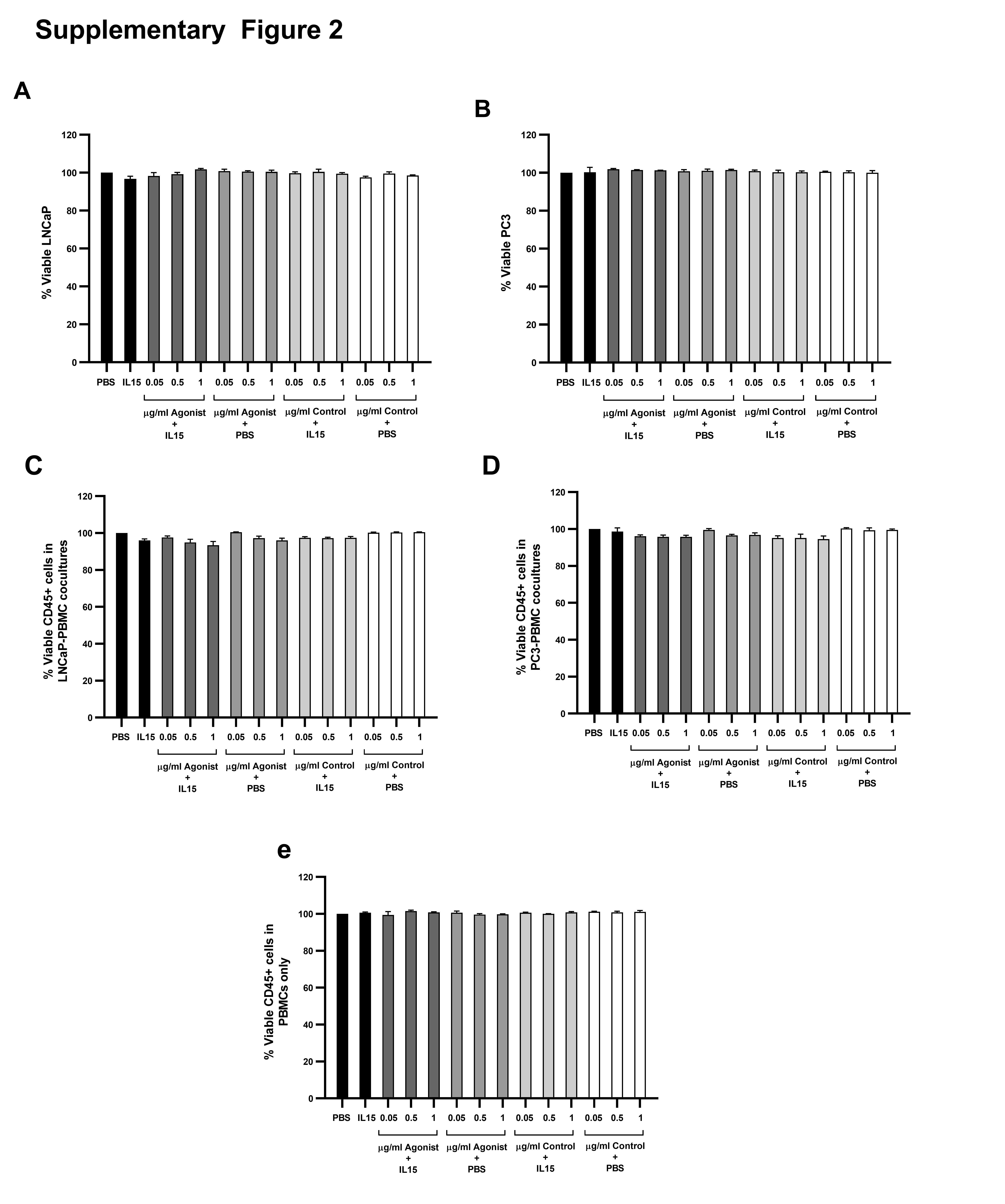

Supplement: Supplementary Figure 2 — Viability of cancer cells without the presence of non-adherent PBMCs, and non-adherent PBMCs with and without the presence of cancer cells after incubation for 48 h in the presence of IL-15 (2.5 ng/ml) or a mixture of IL-15 with different concentrations of the STING agonist 2’3’-c-di-AM(PS)2(Rp/Rp) (ADU-S100 analog- designated as Agonist). Panel (A) shows LNCaP and Panel (B) shows PC3 cancer cells treated with the agents in the absence of PBMCs. Panel (C) and panel (D) show viability of CD45+ cells (non-adherent PBMCs) in the presence of LNCaP and PC3 cells respectively. Panel (E) shows viability of CD45+ cells cultured in the absence of cancer cells. Controls were carried out by replacing IL-15 with PBS and the agonist with a linear nucleotide (2’5’-GpAp), herein designated as Control. Results are means +/− SEM of triplicate or quadruplicate experiments. [file Image_2.jpg]
