# Supplementary material for: Combination of Interleukin-15 With a STING Agonist, ADU-S100 Analog: A Potential Immunotherapy for Prostate Cancer
Source: Front Oncol. 2021 Mar 10;11:621550. doi: 10.3389/fonc.2021.621550 (PMC7988118; doi:10.3389/fonc.2021.621550)
Supplement: Supplementary Table 1 — Antibodies and fluorophores used on this study for flow cytometry. [file Table_1.docx]

**Supplementary Table 1**

| **Antibody** | **Species** | **fluorophore** | **vendor** | **dilution** | **Clone** |
| --- | --- | --- | --- | --- | --- |
| CD45 | Mouse anti-human | FITC | Biolegend | 50x | HI30 |
| CD56 | Mouse anti-human | APC | Biolegend | 50x | MEM-188 |
| CD4 | Mouse anti-human | FITC | Biolegend | 50x | A161A1 |
| CD8 | Mouse anti-human | PERCP.CY5.5 | Biolegend | 50x | SK1 |
| CD8 | Mouse anti-human | FITC | Biolegend | 50x | SK1 |
| CD3 | Mouse anti-human | Pacific Blue | Biolegend | 50x | UCHT1 |
| CD69 | Mouse anti-human | PE | Biolegend | 50x | IV A91 |
| CD80 | Mouse anti-human | PE | Biolegend | 50x | 2D10 |
| CD19 | Mouse anti-human | APC | Biolegend | 50x | 4G7 |
| CD19 | Mouse anti-human | FITC | Biolegend | 45x | 4G7 |
| CD16 | Mouse anti-human | PERCP | Biolegend | 50x | 3G8/B73.1 |
| CD11c | Mouse anti-human | PE | Biolegend | 50X | 3.9/Bu15 |
| CD11c | Mouse anti-human | FITC | Biolegend | 50x | 3.9/Bu15/S-HCL-3 |
| Perforin | Mouse anti-human | PE | Biolegend | 50x | B-D48 |
| CD107a | Mouse anti-human | APC | Biolegend | 50x | H4A3 |
| NKG2D | Mouse anti-human | PECy7 | Biolegend | 50x | 1D11 |
